# Cold-Induced Lipoprotein Clearance in *Cyp7b1*-Deficient Mice

**DOI:** 10.3389/fcell.2022.836741

**Published:** 2022-04-11

**Authors:** Ioannis Evangelakos, Anastasia Kuhl, Miriam Baguhl, Christian Schlein, Clara John, Julia K. Rohde, Markus Heine, Joerg Heeren, Anna Worthmann

**Affiliations:** ^1^ Department of Biochemistry and Molecular Cell Biology, University Medical Center Hamburg-Eppendorf, Hamburg, Germany; ^2^ Institute of Human Genetics, University Medical Center Hamburg-Eppendorf, Hamburg, Germany

**Keywords:** brown adipose tissue, lipoproteins, bile acids, postprandial metabolism, CYP7B1

## Abstract

Brown adipose tissue (BAT) has emerged as an appealing therapeutic target for cardio metabolic diseases. BAT is a heat-producing organ and upon activation substantially lowers hyperlipidemia. In response to cold exposure, not only the uptake of lipids into BAT is increased but also the *Cyp7b1*-mediated synthesis of bile acids (BA) from cholesterol in the liver is triggered. In addition to their role for intestinal lipid digestion, BA act as endocrine signals that can activate thermogenesis in BAT. When exposed to cold temperatures, *Cyp7b1* ^*−/−*^ mice have compromised BAT function along with reduced fecal bile acid levels. Here, we aim to evaluate the role of *Cyp7b1* for BAT-dependent lipid clearance. Using metabolic studies with radioactive tracers, we show that in response to a cold stimulus, BAT-mediated clearance of fatty acids derived from triglyceride-rich lipoproteins (TRL), and their remnants are reduced in *Cyp7b1* ^*−/−*^ mice. The impaired lipid uptake can be explained by reduced BAT lipoprotein lipase (LPL) levels and compromised organ activity in *Cyp7b1* ^*−/−*^ mice, which may be linked to impaired insulin signaling. Overall, our findings reveal that alterations of systemic lipoprotein metabolism mediated by cold-activated BAT are dependent, at least in part, on CYP7Β1.

## Introduction

Activation of thermogenic adipocytes in brown adipose tissue (BAT) and white adipose tissue (WAT) has emerged as a promising therapeutic approach to reduce obesity and associated disorders (Kusminski et al., 2016). Upon sympathetic activation, for example, by a cold stimulus, the expression of uncoupling protein 1 (Ucp1) is increased in brown or beige thermogenic adipocytes in order to uncouple the respiratory chain from ATP synthesis and produce heat instead (Cannon and Nedergaard, 2004). This so-called adaptive thermogenesis enhances global energy expenditure. To meet the high need for energy, BAT takes up huge amounts of lipids and glucose from the circulation and thus substantially contributes to systemic metabolism. In particular, BAT has not only been shown to improve glucose tolerance and insulin resistance (Stanford et al., 2013; Tsagkaraki et al., 2021) but also shown to reduce atherosclerotic burden in pathogenic mouse models (Berbee et al., 2015; Bartelt et al., 2017) most likely due to its strong impact on systemic cholesterol metabolism (Berbee et al., 2015; Bartelt et al., 2017; Worthmann et al., 2017; Schaltenberg et al., 2021). Furthermore, BAT activation corrects hyperlipidemia (Bartelt et al., 2011) by stimulating the liberation and subsequent uptake of fatty acids from triglyceride-rich lipoproteins (TRL) into BAT and promoting the uptake of cholesterol-rich remnant particles into the liver (Berbee et al., 2015; Khedoe et al., 2015). Moreover, in response to cold, BAT internalizes the entire TRL (Fischer et al., 2021).

Bile acids (BA) are produced in the liver from cholesterol and facilitate the ingestion of lipids in the intestine (Russell, 2003). Additionally, recent studies have highlighted the role of BA as endocrine regulators of glucose metabolism (Ahmad et al., 2019; Zheng et al., 2021), satiety (Perino et al., 2021), and obesity (Castellanos-Jankiewicz et al., 2021). Moreover, BA are known to enhance energy expenditure in thermogenic adipose tissues (Watanabe et al., 2006; Velazquez-Villegas et al., 2018) *via* the G-protein-coupled bile acid receptor, Gpbar1, which is also known as TGR5 (Maruyama et al., 2002; Kawamata et al., 2003). In BAT, BA-mediated ligation of TGR5 activates protein kinase A (PKA) and ultimately results in higher expression of *Dio2*, which converts inactive thyroxine (T4) into thyroid hormone (T3) to further stimulate energy expenditure. In addition to the classical synthesis of BA by cytochrome P450 family 7 subfamily A member 1 (CYP7A1), BA are also produced in an alternative fashion by the action of cytochrome P450 family 7 subfamily B member 1 (CYP7B1) (Russell, 2003). In mice, loss of *Cyp7b1* resulted in increased levels of oxysterols (Li-Hawkins et al., 2000), which are precursors of BA. Furthermore, *Cyp7b1* deficiency has been linked to the development of liver steatosis and inflammation (Setchell et al., 1998; Dai et al., 2014; Kakiyama et al., 2020; Evangelakos et al., 2021). Interestingly, our group has recently shown that the activation of BAT by cold not only stimulates the cholesterol flux to the liver but also induces the expression of hepatic *Cyp7b1*, resulting in higher BA production (Worthmann et al., 2017). Additionally, we demonstrated that BAT activity was dependent on CYP7B1 as *Cyp7b1* ^*−/−*^ mice were characterized by reduced BA levels, decreased *Dio2* expression in BAT, and diminished energy expenditure when exposed to cold temperatures. Given the pivotal role of active BAT for lipid metabolism and lipoprotein clearance and also the impact of BA for lipid ingestion, the overall aim of this study was to investigate how the loss of *Cyp7b1* would affect systemic postprandial lipoprotein metabolism when BAT is activated by cold temperatures. Here, we show that in response to cold temperatures, *Cyp7b1* ^*−/−*^ mice have lower systemic BA levels and compromised BAT activation. Further, using radio-active tracer studies, we demonstrate that the BAT-dependent increased processing and clearance of TRL is diminished in *Cyp7b1* ^*−/−*^ mice. Mechanistically, we could link this to decreased lipoprotein lipase (LPL)-dependent processing and an uptake of TRL which may depend on insulin resistance and compromised BAT activity in *Cyp7b1* ^*−/−*^ mice.

## Materials and Methods

### Experimental Animals, Housing Conditions, Diets, and Animal Experiment

All animal experiments were conducted in accordance with FELASA guidelines and approved by the Animal Welfare Officers of the University Medical Center Hamburg-Eppendorf (UKE) as well as the Behörde für Gesundheit und Verbraucherschutz Hamburg (animal protocol 15/96, approved 08 October 2015). Male *Cyp7b1* ^*−/−*^ mice (Li-Hawkins et al., 2000) and littermate control WT mice (C57BL/6J background) were bred and housed in the animal facility of the UKE at 22°C with a day and night cycle of 12 h and *ad libitum* access to food and water. Starting at weeks 12–14 of age, mice were randomized based on body weight, housed in single cages, and fed a Western-type diet (WTD) containing 21% total fat and 0.2% cholesterol (ssniff EF R/M acc. TD88137 mod.) in order to stimulate cold-induced BA synthesis (Worthmann et al., 2017) with *ad libitum* access to water. After a 3-day acclimatization period to the WTD, the mice were either exposed to thermoneutral (30°C) or cold (6°C) housing conditions for 1 week in total. Single caging was performed to estimate daily food intake and to avoid group cuddling and warming during the cold housing intervention. Organ harvests and metabolic turnover studies were performed after a 4-h fasting period, and the mice were anesthetized with a lethal dose of ketamine and xylazine. Cardiac blood was drawn with syringes containing 5 μl 0.5 M EDTA. Animals were perfused with PBS containing 10 U/ml heparin, and then the organs were harvested and immediately stored at −80°C for further analysis.

### Plasma Analysis

Plasma was generated by a 10-min centrifugation of the EDTA-spiked blood at 10,000 g and 4°C in a benchtop centrifuge. Plasma triglycerides were determined using a commercial kit (Roche, Mannheim, Germany) adapted to a 96-well plate format with Precipath® (Roche) as a standard. Plasma insulin levels were measured with an ELISA kit (#90060, Chrystal Chem, Zaandam, Netherlands) according to the manufacturer’s protocol following a low range detection assay. Bile acid analysis was carried out by HPLC coupled to electrospray ionization tandem mass spectrometry as described before (Wegner et al., 2017). Briefly, plasma was spiked with internal standards, and then BA were extracted using methanol. Quantitative measurement of BA was performed using an LC-ESI-QqQ system run on the multiple reaction monitoring (MRM) mode: HPLC: NEXERA X2 LC-30AD HPLC PUMP (Shimadzu); column: Kinetex C18 (100 Å, 150 × 2.1 mm i.d, Phenomenex); QqQ: Q trap 5500 System (SCIEX). Peak identification and quantification were performed by comparing retention times as well as MRM transitions and peak areas, respectively, to particular corresponding standard chromatograms.

### Metabolic Turnover Studies

For the oral fat tolerance test (OFTT), mice were fasted for 4 h at zeitgeber time (ZT) 1 and subsequently gavaged at ZT5 with 200 µl corn oil containing ^3^H-triolein (185 kBq/mouse) and ^14^C cholesterol (37 kBq/mouse). Blood was taken from the tail vein before (ZT5), 60 min (ZT6), and 120 min (ZT7) after the gavage to monitor blood radioactivity by scintillation counting (see later). For the chylomicron production, mice were fasted for 4 h at ZT1 and tail vein-injected with 150 µl Triton WR-1339 solution (5 g/kg body weight), followed by direct gavage of 200 µl corn oil (ZT5) containing ^3^H-triolein (185 kBq/mouse) and ^14^C cholesterol (30 kBq/mouse). Tail vein blood was taken before (ZT5), 60 (ZT6), 120 (ZT7), and 240 (ZT9) min after the gavage to monitor blood radioactivity by scintillation counting (see later). Depending on the turnover study, mice were anaesthetized after 2 or 4 h, and in all cases, the organs were harvested as described previously. Scintillation counting was performed using a Perkin Elmer Tricarb Scintillation Counter, either directly for plasma or after dissolving the organs in 10x (v/m) Solvable™ by shaking at 60°C.

### Gene Expression Analysis

Tissues were lysed in TRIzol (Ambion, Life Technologies) using a TissueLyser (Qiagen). Nucleic acids were extracted with chloroform, and total RNA was isolated using the RNA purification kit NucleoSpin®RNA II (Macherey & Nagel). RNA concentration was determined with NanoDrop, and 400 ng of RNA was used for reverse transcription into cDNA by using the III Reverse Transcriptase (Invitrogen, Waltham, MA, United States). Quantitative real-time PCR was performed either on an ABI 7900HT or a QuantStudio™ 5 Real-Time PCR System (Applied Biosystems) using TaqMan on-demand primer sets (Applied Biosystems, Waltham, MA, United States) (*Acaca*: Mm01304285_m1, *Angptl4*: Mm00480431_m1, *CD36*: Mm00432403_m1, *Chrebp-beta*: AIVI4CH, *Cyp27a1*: Mm00470430_m1, *Cyp7a1*: Mm00484150_m1, *Cyp7b1*: Mm00484157_m1, *Cyp8b1*: Mm00501637_s1, *Fasn*: Mm00662319_m1, *Gpihbp1*: Mm01205849_g1, *Lipe*: Mm00495359_m1, *Lipg*: Mm00495368_m1, *Lpl*: Mm00434764_m1, *Pnpla2*: Mm00503040_m1, Scd1: Mm00772290_m1, *Slc27a1*: Mm00449511_m1, *Slc27a4*: Mm01327405_m1, *Slc27a5*: Mm00447768_m1, *Slc2a1*: Mm00441480_m1, *Slc2a4*: Mm01245502_m1). mRNA levels were normalized to the level of the housekeeping gene TATA-box binding protein (Tbp) mRNA, and the results were displayed as relative gene expression normalized to the experimental control group, following calculations using the 2^-ΔΔCt^ method.

### Western Blot Analysis

For SDS–PAGE, 10x excess of (v/w) RIPA buffer (50 mM Tris–HCl pH 7.4, 5 mM EDTA, 150 mM sodium chloride, 1 mM sodium pyrophosphate, 1 mM sodium fluoride, 1 mM sodium ortho-vanadate, 1% (NP-40) supplemented with cOmplete mini protease inhibitor cocktail tablets (Roche), and phosphatase inhibitor cocktail (Bimake.com) was added to tissues which were subsequently homogenized in a tissue lyser (Qiagen). After centrifugation at 16,000 g for 10 min, the clear soluble middle layer of the lysate was taken, and protein concentration was assessed using the method of Lowry. Then 20 µg of total protein was denatured at 55°C for 10 min in a NuPAGE reducing sample buffer (Invitrogen) and separated on 10% SDS–polyacrylamide Tris–glycine gels. Proteins were transferred to nitrocellulose membranes in a wet blotting system. Equal loading was confirmed by Ponceau S (Serva) staining. Subsequently, the membranes were washed twice in TBS-T (20 mM Tris, 150 mM sodium chloride, 0.1% (v/v) Tween 20) and blocked for 1 h in 5% milk powder (Sigma) in TBS-T at room temperature. Primary antibodies were incubated (5% BSA in TBS-T) overnight at 4°C, and secondary antibodies were diluted in 5% milk powder in TBS-T. Detection was performed with enhanced chemiluminescence using an Amersham Imager 600 (GE Healthcare). The following primary antibodies were used: γ-tubulin (rabbit monoclonal; 1:2,000; cat. No. ab179503, Abcam), AKT (rabbit polyclonal; 1:1,000, cat#9272, Cell Signaling), phospho-AKT-(Ser473) (rabbit polyclonal; 1:1,000, cat#9272, Cell Signaling), LPL (goat polyclonal; 1:2,000, kind gift from Stephen G. Young (Page et al., 2006)), FASN (mouse monoclonal; 1:1,000, cat#610962BD, Biosciences), phospho-(Ser/Thr) PKA substrate (rabbit polyclonal; 1:1,000, cat#9621, Cell Signaling), total OXPHOS cocktail (mouse monoclonal; 1:25,000, cat#ab110413, Abcam), GPIHBP1 (rat monoclonal; 1:1000, a kind gift from Stephen G. Young (Beigneux et al., 2009)). The following secondary antibodies, in a dilution of 1:5,000, were used: HRP goat anti-rabbit (cat# 111-035-144, Jackson ImmunoResearch Labs), HRP goat anti-mouse (cat#115-035-003, Jackson ImmunoResearch Labs), HRP donkey anti-rat (cat#712-035-150, Jackson ImmunoResearch Labs), and HRP mouse anti-goat (cat# 205-035-108, Jackson ImmunoResearch Labs).

### Triiodothyronine (T3) ELISA Measurement

T3 levels in BAT were detected using a commercial ELISA T3-kit (T3043T-100, CALBIOTECH). Tissue extracts were prepared in a lysis buffer (100 mM Tris pH 7.4, 150 mM NaCl, 1 mM EGTA, 1 mM EDTA, 1% Triton X-100, and 0.5% sodium deoxycholate) supplemented with cOmplete mini protease inhibitor cocktail tablets (Roche). After homogenization in tissue lyser (Qiagen), samples were maintained under constant agitation for 2 h at 4°C. Supernatants, containing the soluble protein extract, were obtained by centrifugation for 20 min at 16,000 g. Subsequent T3 detection and calculations were done following the manufacturer’s protocol.

### Statistics

Data are expressed as mean ± SEM. Student’s t-test and one- or two-way ANOVA in combination with post hoc correction for multiple testing (Tukey test) were used for comparison between the groups. GraphPad Prism 7.0 was used for statistical calculations and the ABC system to depict significant differences, where different letters indicate statistical significance with *p* < 0.05.

## Results

### *Cyp7b1* Depletion Impairs Cold-Induced Bile Acid Synthesis and Affects Brown Adipose Tissue Responses

**FIGURE 1:**
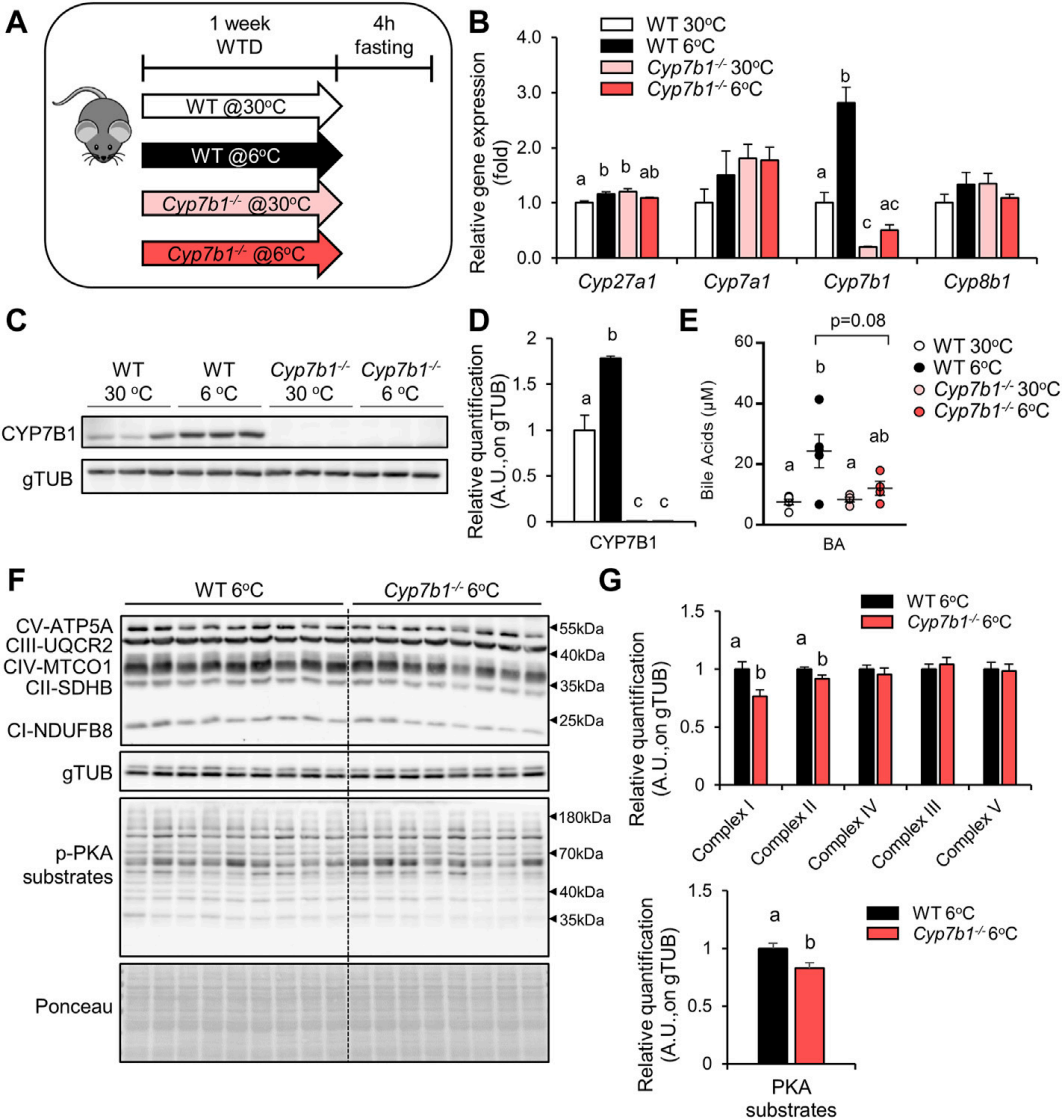
Deletion of *Cyp7b1* attenuates the cold-induced bile acid (BA) synthesis and correlates with compromised brown adipose tissue (BAT) responses. Wild-type (WT) and *Cyp7b1* ^*−/−*^ littermates were fed a Western-type diet (WTD) and housed for 1 week either at a thermoneutral (30°C) or cold (6°C) environment. **(A)** Experimental setup, **(B)** hepatic gene expression of bile acid synthesis genes, and **(C)** hepatic protein levels of CYP7B1 **(D)** with relative quantification and **(E)** circulating BA levels (n = 3-4/group). **(F)** Representative Western blotting of OXPHOS complexes and pPKA substrates **(G)** with relative quantification of cold-exposed WT (n = 9) and *Cyp7b1* ^*−/−*^ (n = 8) mice. Data are shown as mean values ±SEM. Different letters indicate significant differences between groups (*p* < 0.05) determined by two-way ANOVA.

Once activated by cold, BAT has been shown to internalize substantial amounts of lipids and glucose, thus attenuating hyperlipidemia (Bartelt et al., 2011) and hyperglycemia (Stanford et al., 2013). In previous studies, we found that *Cyp7b1*-mediated BA synthesis is upregulated in the livers of cold-housed mice and that *Cyp7b1* is critical for BAT activity (Worthmann et al., 2017). Here, we aimed to investigate the effects of *Cyp7b1* deficiency for BAT-mediated lipid handling. For this purpose, we housed wild-type (WT) and *Cyp7b1* ^*−/−*^ mice at thermoneutral (30°C) or cold (6°C) conditions to activate BAT (Figure 1A). In line with our previous report, we observed a significant upregulation of hepatic *Cyp7b1* expression in WT mice kept at 6°C, while very low *Cyp7b1* transcripts were detected in the *Cyp7b1* ^*−/−*^ mice both at 30°C and at 6°C (Figure 1B). The expression of *Cyp27a1*, another critical component of the alternative BA synthesis pathway, was higher in WT mice after cold exposure and also higher in *Cyp7b1* ^*−/−*^ mice (Figure 1B). Other mediators of BA synthesis (*Cyp7a1* and cytochrome P450, family 8, subfamily B, polypeptide 1 = *Cyp8b1*) were neither affected by cold nor by *Cyp7b1* deficiency on a transcript level (Figure 1B). In agreement, CYP7B1 protein levels were higher in cold-exposed WT mice, while no protein was detected in the knockout mice (Figures 1C,D). Importantly, in WT mice, higher hepatic *Cyp7b1* expression upon cold housing also translated into higher plasma BA levels, an effect that was blunted in *Cyp7b1* ^*−/−*^ mice (Figure 1E). Of note, cold housing did not affect BA pool composition (data not shown) but resulted in an overall increase in BA levels throughout all BA species (Supplementary Figure S1), which was reversed in *Cyp7b1* ^*−/−*^ mice (Supplementary Figure S1). As BA have been shown to activate BAT *via* Tgr5 (Watanabe et al., 2006; Broeders et al., 2015), and we detected higher levels of the TGR5 agonists TDCA/TCDCA after cold housing in WT mice but not in *Cyp7b1* ^*−/−*^ mice (Supplementary Figure S1), we next explored levels of phosphorylated PKA substrates (pPKA substrates) in iBATs of *Cyp7b1* ^*−/−*^ mice after cold housing. As expected and in agreement with our recent report that upon cold housing, *Cyp7b1* ^*−/−*^ mice displayed decreased energy expenditure and diminished thermogenic markers at the transcriptional (*Ucp1*, *Ppargc1a*, *Elovl3*, and *Dio2*) and protein levels (UCP1) (Worthmann et al., 2017); we found reduced levels of pPKA substrates mostly attributed to lower intensities of proteins in the range of 40 kDa (Figures 1F,G). In particular, the phosphorylation of CREB, the crucial mediator of PKA signaling, was reduced by trend in *Cyp7b1* ^*−/−*^mice upon cold exposure (Supplementary Figure S2A). In line, T3 levels were lower in BAT from cold-housed *Cyp7b1* ^*−/−*^ mice (Supplementary Figure S2B). Additionally, we detected altered protein levels of OXPHOS complexes in cold-housed *Cyp7b1* ^*−/−*^ mice (Figures 1F,G). While complexes III–V were not affected, we observed a significant lower abundance of complex I and II (Figures 1F,G). In agreement with our previous report, these data support that *Cyp7b1* deletion and reduced BA levels compromise BAT thermogenic responses, which in turn might affect postprandial lipid metabolism.

### *Cyp7b1* Depletion Diminishes BAT-Mediated Lipoprotein Clearance in Response to Cold Treatment

**FIGURE 2:**
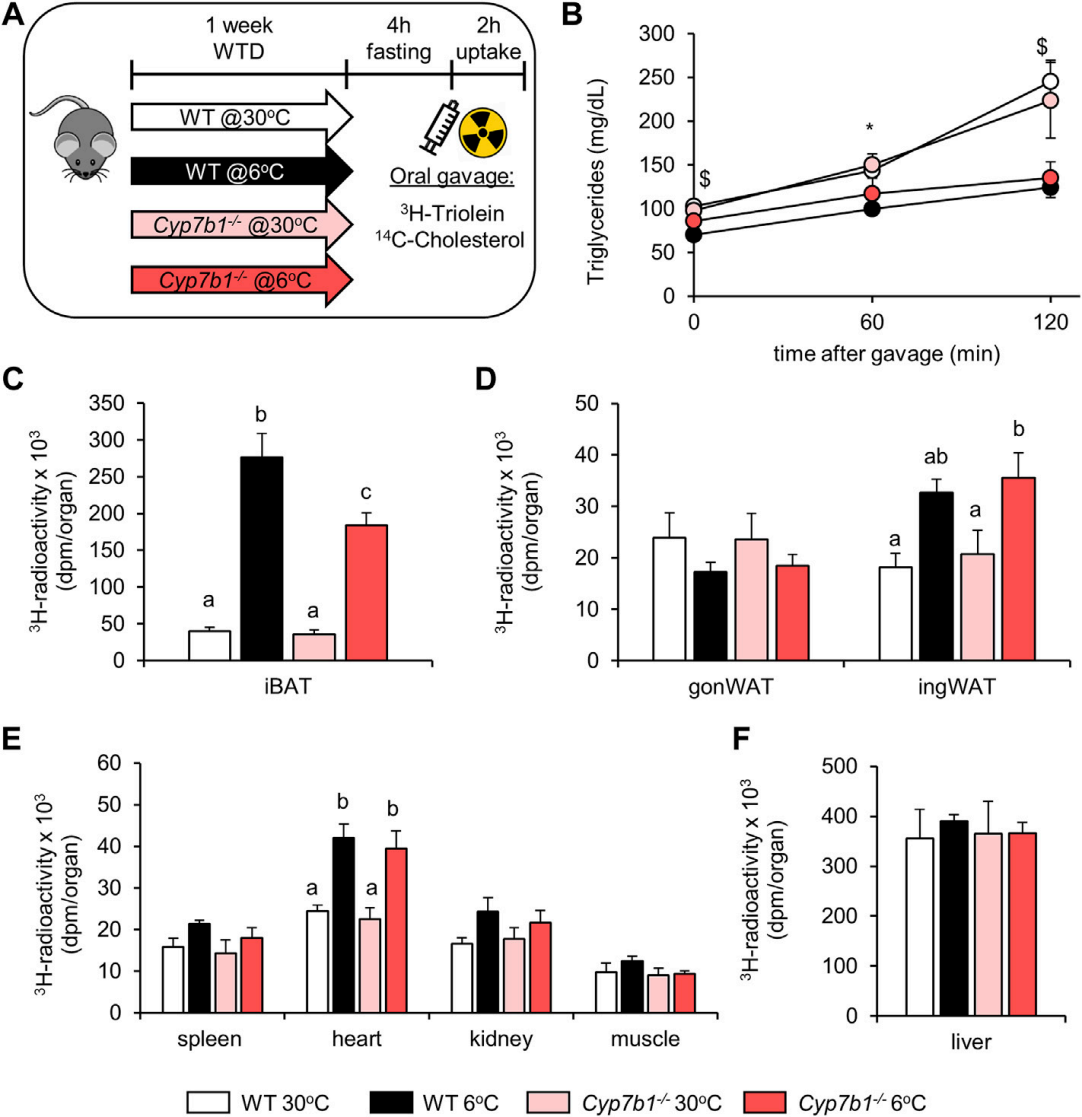
*Cyp7b1* deletion compromises cold-induced fatty acid uptake into BAT. WT and *Cyp7b1* ^*−/−*^ littermates fed a WTD were housed for 1 week either at thermoneutral (30°C) or cold (6°C) environment before being subjected to a fat tolerance test (OFTT) with ^3^H-triolein/^14^C-cholesterol-traced oral gavage. Experimental setup (applies also to Figure 3) **(A)**, plasma triglyceride levels **(B)** and total uptake of ^3^H-triolein in brown **(C)** and white **(D)** adipose tissue depots as well as other metabolic organs **(E**,**F)** (n = 5/group). Data are shown as mean values ±SEM. For **(B)**, *p* values lower than 0.05 were considered statistically significant and are indicated as follows: $ = WT30 vs. WT 6; * = WT 6 vs. KO 30; # = WT 30 vs. KO 6; and & = KO 30 vs. KO 6. For **(C–E)**, different letters indicate significant differences between groups (*p* < 0.05) determined by two-way ANOVA.

**FIGURE 3:**
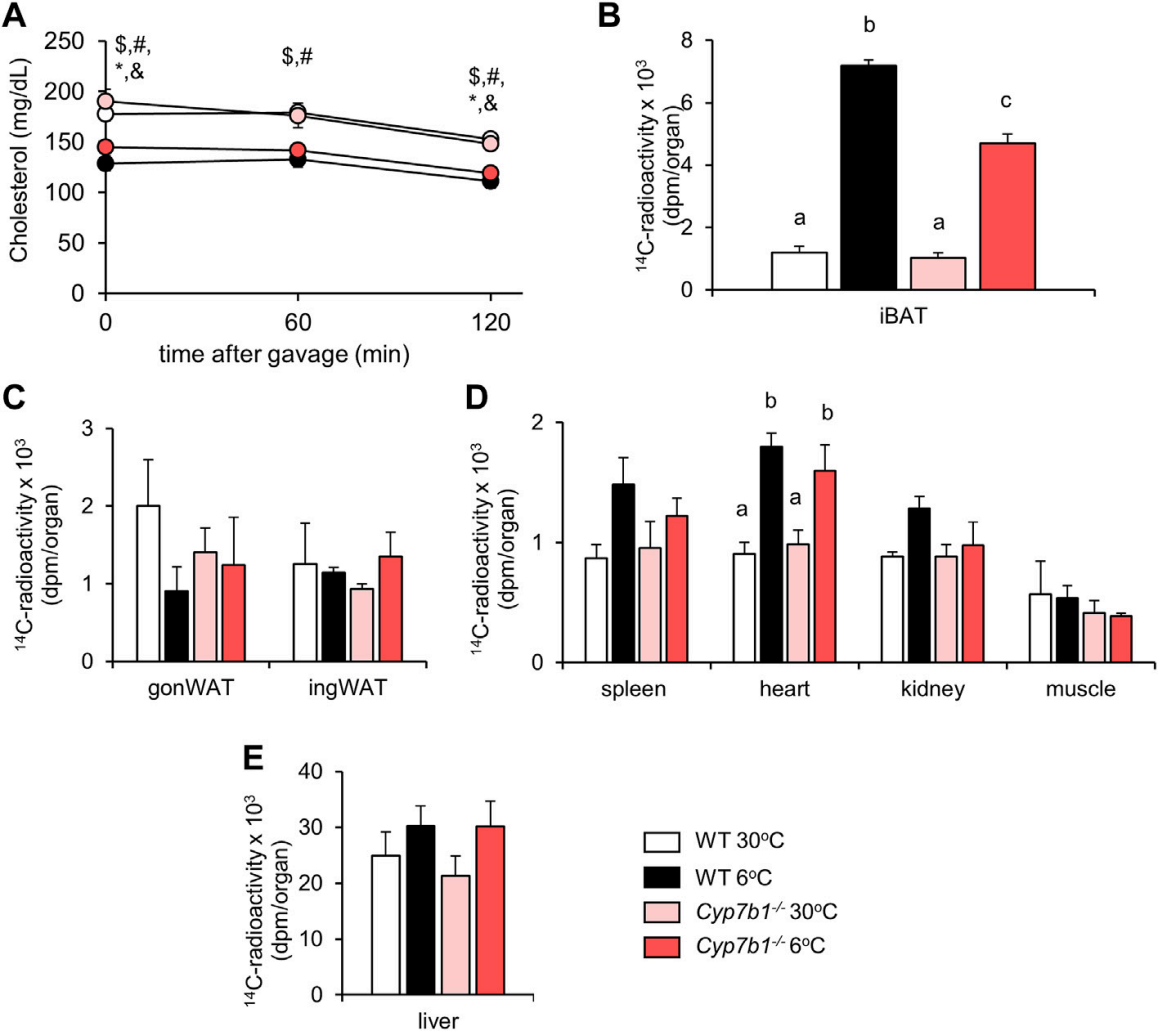
*Cyp7b1* deletion compromises cold-induced chylomicron remnant uptake into BAT. WT and *Cyp7b1* ^*−/−*^ littermates fed a WTD were housed for 1 week either at thermoneutral (30°C) or cold (6°C) environment before being subjected to a fat tolerance test (OFTT) with ^3^H-triolein/^14^C-cholesterol-traced oral gavage (same experimental setup with Figure 2). Plasma cholesterol levels **(A)**, plasma triglyceride levels, total uptake of ^14^C-cholesterol in brown **(B)** and white **(C)** adipose tissue depots as well as other metabolic organs **(D, E)** (n = 5/group). Data are shown as mean values ±SEM. For Panel **(A)** *p* values lower than 0.05 were considered statistically significant and are indicated as follows: $ = WT30 vs. WT 6; * = WT 6 vs. KO 30; # = WT 30 vs. KO 6; & = KO 30 vs. KO 6; For panel **(B–E)**: Different letters indicate significant differences between groups (*p* < 0.05) determined by two-way ANOVA.

After prolonged sympathetic activation, thermogenic adipose tissues significantly contribute to lipoprotein metabolism (Bartelt et al., 2011; Bartelt et al., 2017; Worthmann et al., 2017). We speculated that decreased BAT activity observed in *Cyp7b1* ^−/−^ mice would compromise BAT-mediated lipoprotein clearance. To explore whether reduced bile acid levels detected in *Cyp7b1* ^*−/−*^ mice influence lipid disposal in cold-challenged mice, we performed an oral fat tolerance test in WT and *Cyp7b1* ^−/−^ mice housed at 30°C or 6°C. After fasting for 4 h, we orally administered corn oil spiked with radioactively labeled ^3^H-triolein and ^14^C-cholesterol and monitored blood clearance for 120 min and organ uptake (Figure 2A). Of note, by using these radiotracers, we were able to follow vascular lipoprotein processing and the uptake of free fatty acids (^3^H-label) as well as the uptake of core lipoproteins (^14^C-label) at the same time. As expected, in all groups, plasma triglyceride (TG) levels increased during the time of the experiment as a postprandial response (Figure 2B). In line with previous findings, while the TG levels doubled over time in both warm-housed groups, the increases were only moderate in cold-housed mice. After 120 min, plasma TG levels were significantly lower in cold-housed mice than in warm-housed mice irrespective of the genotype (Figure 2B). Interestingly, we detected higher amounts ^3^H-radioactivity in the iBAT of cold-housed WT mice than their warm counterparts, while this effect was significantly diminished in *Cyp7b1* ^−/−^ mice (Figure 2C). However, compared to both warm-housed groups, the amount of ^3^H-triolein-derived fatty acids in iBAT was still higher in cold-housed *Cyp7b1* ^−/−^ mice (Figure 2C). Irrespective of the genotype, cold treatment enhanced the uptake of ^3^H-labeled fatty acids into inguinal WAT (ingWAT) depots (Figure 2D). These data indicate that in BAT of *Cyp7b1* ^−/−^ mice, the cold-induced increase in vascular lipoprotein processing and subsequent fatty acid uptake into thermogenic adipocytes are compromised. Uptake of triolein-derived fatty acids into the heart (Figure 2E) was enhanced after cold-treatment but not affected by a loss of *Cyp7b1*, while it remained unaffected in the gonWAT (Figure 2D), the spleen, kidneys, muscles (Figure 2E), and the liver (Figure 2F). Levels of plasma cholesterol remained similar before and 120 min after the oil-gavage in all groups (Figure 3A). Of note, in agreement with recent reports, total plasma cholesterol levels in cold-housed mice were significantly lower than in warm-housed mice. This was not affected by the absence of *Cyp7b1* (Figure 3A). In line with what we observed for the ^3^H-label, we detected significantly higher amounts of ^14^C-cholesterol in iBAT of both WT and *Cyp7b1* ^−/−^ mice (Figure 3B). However, ^14^C-tracer levels were reduced in cold-housed *Cyp7b1* ^−/−^ mice compared to their WT littermates (Figure 3B). In accordance with the uptake of radiolabeled fatty acids, cholesterol uptake into other organs (ingWAT, gonWAT, spleen, kidneys, muscles, and liver) remained unchanged (Figures 3C–E). Again, irrespective of the genotype, we detected increased amounts of ^14^C-cholesterol in the heart of cold-exposed mice (Figure 3D). Altogether, these data suggest that the uptake of fatty acids (^3^H-label) and entire lipoprotein-core remnants (^14^C-label) into iBAT is attenuated in cold-housed *Cyp7b1* ^*−/−*^ mice.

### *Cyp7b1* Depletion Does Not Affect Intestinal Lipoprotein Secretion in Response to Cold Treatment

**FIGURE 4:**
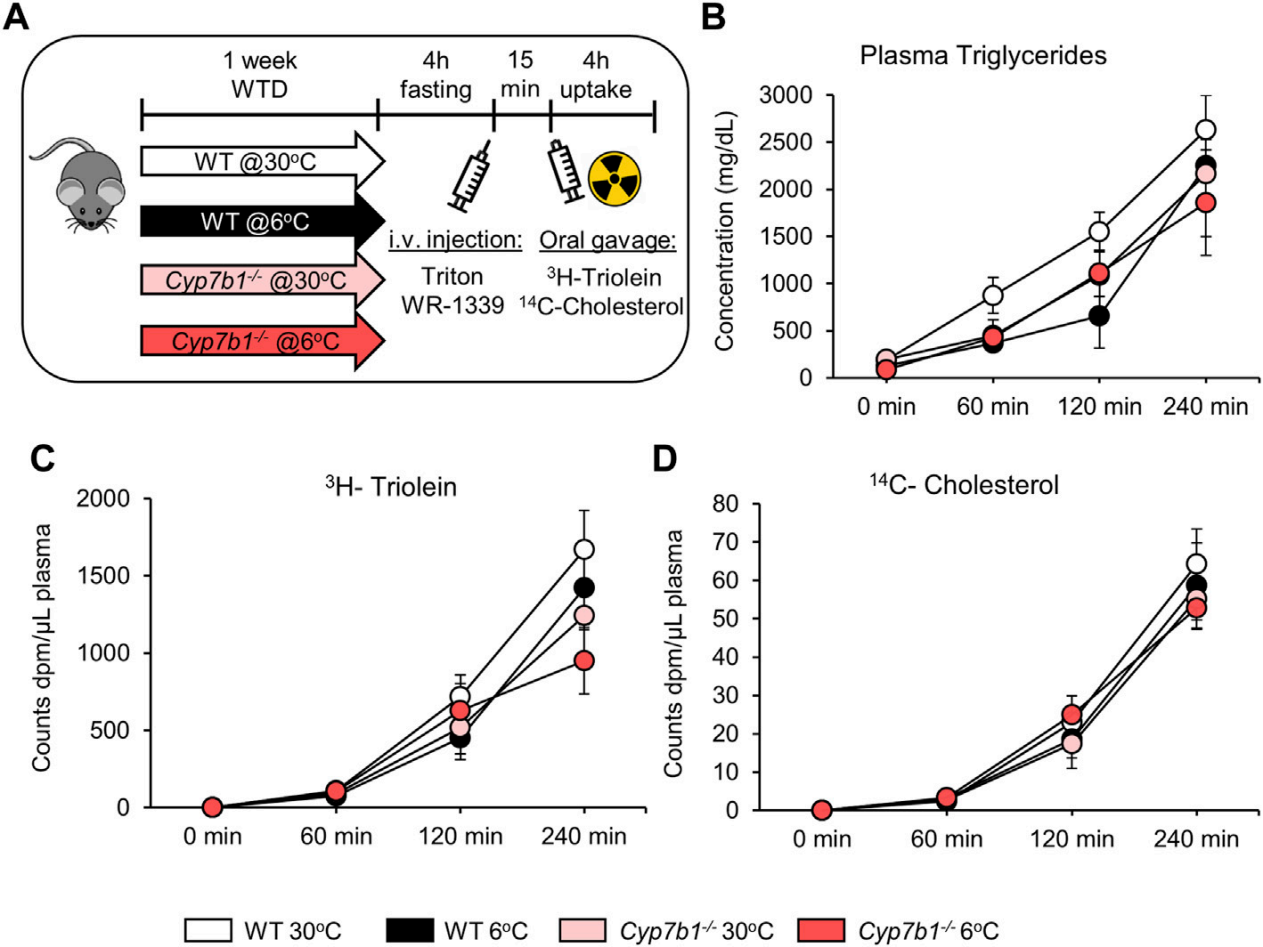
*Cyp7b1* deletion does not affect intestinal chylomicron production in response to cold exposure. WT and *Cyp7b1* ^*−/−*^ littermates were fed a WTD and housed for 1 week either at a thermoneutral (30°C) or cold (6°C) environment. Then 15 min before the ^3^H-triolein/^14^C-cholesterol-traced lipid gavage, mice were intravenously injected with Triton WR-1339 to inhibit intravascular lipoprotein hydrolysis and follow the accumulation of intestinal secreted chylomicrons. Experimental setup **(A)**, triglyceride **(B)**, ^3^H-triolein **(C)**, and ^14^C-cholesterol **(D)** levels in the plasma between 0 and 240 min post-gavage (n = 5/group). Data are shown as mean values ±SEM. Different letters indicate significant differences between groups (*p* < 0.05) determined by two-way ANOVA.

Postprandial lipoprotein processing not only depends on clearance by metabolic tissues such as adipose tissues, heart, and muscle but also is highly dependent on intestinal lipid uptake and lipoprotein assembly. Of note, especially, lipid ingestion is mediated by BA and might therefore be affected by the depletion of *Cyp7b1*. In order to rule out any effects of *Cyp7b1* on intestinal lipid ingestion and lipoprotein secretion, we administered an oral fat load to the mice in the presence of the detergent Triton WR-1339. This compound inhibits lipases and blocks intravascular hydrolysis of triglyceride-rich lipoproteins (Kellner et al., 1951; Schotz et al., 1957), which leads to their accumulation in the systemic circulation and thus allows the investigation of intestinal chylomicron production. As before, the fat load contained radiolabeled ^3^H-triolein and ^14^C-cholesterol (Figure 4A). As expected, upon blocking intravascular hydrolysis of lipoproteins, plasma triglyceride levels increased dramatically over time and accumulated in a range of 2,000-3,000 mg/dl on a very high level after 240 min (Figure 4B). Of note, at 240 min, we did not detect any significant differences, regardless of the genotype or housing temperature (Figure 4B). In line, plasma levels of ^3^H-triolein and ^14^C-cholesterol also strongly increased over the 4-h period of the experiment, but after 4 h, no differences were detected between the groups (Figures 4C,D). Together, these data indicate that neither cold housing nor loss of *Cyp7b1* affects intestinal chylomicron production. Accordingly, the alterations in postprandial lipoprotein metabolism observed in *Cyp7b1* ^−/−^ mice are rather dependent on BAT-mediated lipoprotein clearance.

### *Cyp7b1* Depletion Alters Machinery for Intravascular Lipoprotein Clearance in Response to Cold Treatment

**FIGURE 5:**
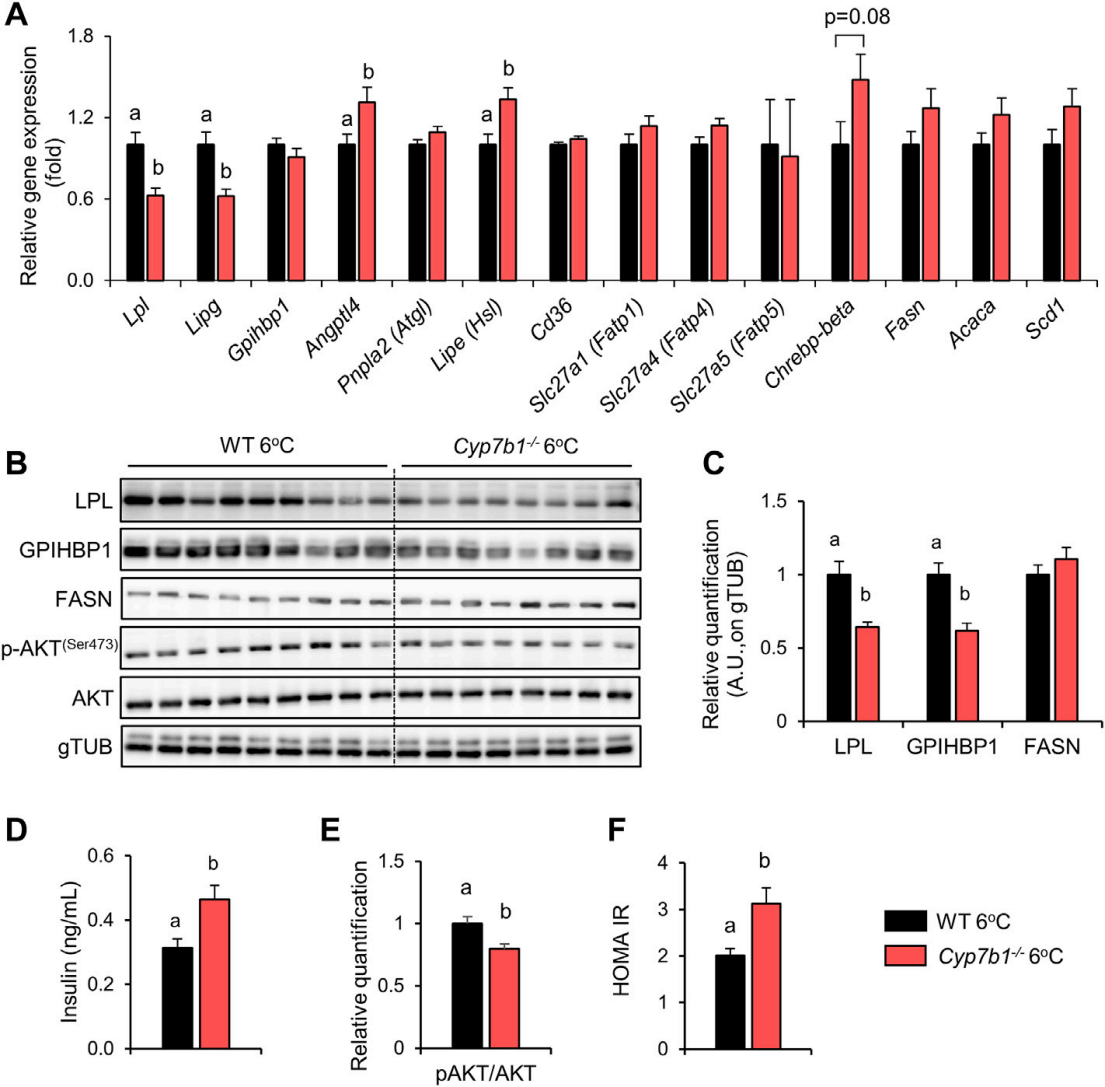
*Cyp7b1* deletion impairs BAT lipoprotein processing responses during cold exposure. WT (n = 9) and *Cyp7b1* ^*−/−*^ (n = 8) littermates were fed a WTD and housed for 1 week at a cold (6°C) environment. BAT relative gene expression of lipoprotein processing/uptake, glucose uptake, and *de novo* lipogenesis markers **(A)**, protein levels of LPL, GPIHBP1, FASN, p-AKT and AKT **(B)** with relative quantification of LPL, GPIHBP1, and FASN **(C)**. Plasma insulin levels **(D)**, relative quantification of p-AKT and AKT blots shown in B **(E)**, and HOMA-IR **(F)**. Data are shown as mean values ±SEM. Different letters indicate significant differences between groups (*p* < 0.05) determined by two-way ANOVA.

To get a deeper understanding on how the loss of *Cyp7b1* may interfere with lipoprotein clearance by BAT mechanistically, we took BAT samples of cold-housed WT and *Cyp7b1* ^−/−^ mice and performed gene expression analysis. In line with the reduced uptake of lipoproteins into BAT of *Cyp7b1* ^−/−^ mice, we detected lower copy numbers of gene-encoding proteins mediating intravascular processing and uptake of lipoproteins. In particular, gene expression (Figure 5A) of the lipases, endothelial lipase (encoded by *Lipg*) and lipoprotein lipase (encoded by *Lpl*), was decreased in *Cyp7b1* ^−/−^mice, while the gene expression of LPL’s negative regulator angiopoietin-like 4 (*Angplt4*) was increased (Figure 5A). This was also translated into reduced LPL protein levels (Figure 5B). In the same line, glycosylphosphatidylinositol-anchored high-density lipoprotein-binding protein 1 (*Gpihbp1*), which is important for intravascular localization of LPL, was not altered on the transcript level (Figure 5A) but significantly reduced on the protein level after loss of *Cyp7b1* (Figures 5B,C). In line with an increased uptake of lipids into the hearts of cold-housed mice irrespective of the genotype (Figure 2E, Figure 3D), cardiac LPL and GPHIBP1 protein levels were not affected by loss of *Cyp7b1* (Supplementary Figure S3). Of note, expression of fatty acid receptors and transport proteins (*CD36*, *Slc27a1*, *Slc27a4*, and *Slc27a5*) (Figure 5A) in BAT remained unchanged. Interestingly, the expression of genes involved in lipolysis (hormone-sensitive lipase encoded by *Lipe* and adipose triglyceride lipase encoded by *Pnpla2*) (Figure 5A) and *de novo* lipogenesis (carbohydrate response element binding protein 1 beta (*Chrebp1b*), fatty acid synthase (*Fasn*) (Figures 5A–C), acetyl-CoA carboxylase alpha (*Acaca*), and stearoyl-CoA desaturase-1 (*Scd1*) (Figure 5A)) trended to be increased as well. We next aimed to explore how lower expression of lipases and especially *Lpl* might be regulated in BAT of cold-housed *Cyp7b1* ^−/−^ mice. As *Lpl* is activated by insulin in BAT, we assessed both plasma insulin levels and iBAT insulin signaling in WT and *Cyp7b1* ^−/−^ mice. We found compared to WT mice, cold-housed *Cyp7b1* ^−/−^ mice had significantly higher levels of plasma insulin (Figure 5D). Additionally, by Western blot, we assessed the levels of insulin-mediated phosphorylation of protein kinase B at serine 473 (pSer473) as a surrogate marker for insulin action. We found significantly reduced levels of pAKT in the BAT of *Cyp7b1* ^−/−^ mice (Figures 5B,E). Moreover, HOMA-IR was higher in cold-exposed *Cyp7b1*-deficient mice (Figure 5F). Thus, despite hyperinsulinemia, insulin signaling was lower in *Cyp7b1* ^−/−^ mice, suggesting that these mice were insulin-resistant. To conclude, upon cold exposure, reduced intravascular lipoprotein processing and hydrolysis in BAT of *Cyp7b1* ^−/−^ mice might be dependent on reduced activity of LPL and other lipases as a result of impaired insulin-mediated activation of LPL.

## Discussion

As thermogenic adipose tissues substantially contribute to systemic lipid metabolism (Bartelt et al., 2011; Worthmann et al., 2017) and were detected to be functional also in humans (Nedergaard et al., 2007; Cypess et al., 2009; Saito et al., 2009; van Marken Lichtenbelt et al., 2009; Virtanen et al., 2009), their activation has given promising results for the treatment of metabolic disorders (O'Mara et al., 2020). Nevertheless, an in-depth understanding of how these processes are regulated and which factors might contribute to the beneficial effects of BAT for metabolic health is inevitable in order to harness thermogenic adipose tissues therapeutically. Our study aimed to further investigate the interplay between BA and BAT. In particular, we focused on the question how the loss of *Cyp7b1* and CYP7B1-derived bile acids might affect BAT-dependent lipoprotein processing. Since BA have been shown to activate BAT and enhance energy expenditure in mice and men (Watanabe et al., 2006; Broeders et al., 2015) in a TGR5-dependent manner (Velazquez-Villegas et al., 2018), we hypothesized that besides reduced fecal BA levels and lower energy expenditure in BAT, *Cyp7b1* ^*−/−*^ mice would have altered lipoprotein clearance when exposed to cold temperatures. In line with our previous report (Worthmann et al., 2017), we found that in response to cold exposure, *Cyp7b1* ^*−/−*^ mice not only have reduced fecal but also systemic BA levels. These translated into reduced BA-mediated BAT activation as assessed by the reduced levels of pPKA substrates. This is in accordance with reduced phosphorylation of cAMP response element-binding protein (pCREB) observed in mice lacking TGR5 specifically in adipocytes (Velazquez-Villegas et al., 2018). We thus speculate that in our model, reductions in the pPKA substrate might stem from attenuated TGR5 signaling. In the same line, lower levels of OXPHOS complex I detected in cold-housed *Cyp7b1* ^*−/−*^ mice may be a result of decreased PKA signaling (Papa et al., 2012) and thus also mediated by the impaired BA-TGR5 axis after the loss of *Cyp7b1*. To directly link *Cyp7b*1 deficiency and lower BA levels with the BAT function, in the future, more detailed studies should be performed in mice lacking the BA receptor TGR5 specifically in thermogenic adipose tissues (such as the *Gpbar1* ^flox/flox^ *Ucp1* cre or *Adipoq* cre). The attenuated activation of BAT in *Cyp7b1* ^*−/−*^ mice indeed affected postprandial metabolism, and in particular the clearance of TRL particles. Here, especially the uptake of TRL remnant particles and TRL-derived fatty acids into the BAT was decreased in the absence of *Cyp7b1*, while similar uptake was observed into the iWAT of cold-housed WT and *Cyp7b1* ^*−/−*^ mice. Irrespective of the genotype, we detected an increased lipid uptake into the hearts of cold-housed mice, which is in line with previous studies reporting cardiac hypertrophy and increased cardiac lipid uptake upon cold acclimation (Cheng and Hauton, 2008). Importantly, by inhibiting the intravascular processing of TRL particles, we could rule out possible effects of *Cyp7b1*- and CYP7B1-derived BA on intestinal lipid ingestion, as BA are important mediators of lipid digestion processes (Kuipers et al., 2014). In this context, especially hydrophilic BA such as muricholates which are produced by CYP2C70 and are predominantly present in mice (Takahashi et al., 2016; de Boer et al., 2020) have been shown to inhibit cholesterol absorption (Wang et al., 2003). Therefore, in mice, alterations in the hydrophilic BA pool either mediated by cold or by inactive CYP7B1-CYP2C70 axis could reasonably affect intestinal cholesterol absorption. However, despite higher systemic BA levels in cold-housed WT mice and reduced BA levels in *Cyp7b1* ^*−/−*^ mice, we did not observe reduced or increased cholesterol absorption, respectively.

In response to cold, BAT not only takes up TRL-derived fatty acids (Bartelt et al., 2011; Berbee et al., 2015; Khedoe et al., 2015) but also entire TRL particles (Bartelt et al., 2011; Fischer et al., 2021), a process which is dependent on insulin (Heine et al., 2018). LPL is an important mediator of TRL hydrolysis (Auwerx et al., 1992) and is transported to the capillary lumen by GPIHBP1 (Davies et al., 2010). In accordance with reduced uptake of fatty acids and TRL-remnant particles, LPL and GPIHBP1 levels were lower in cold-exposed *Cyp7b1* ^*−/−*^ mice. Moreover, ANGPTL4 has been described to be critical for BAT TRL uptake (Dijk et al., 2015), and *Angptl4* transcript levels were increased in cold-housed *Cyp7b1* ^*−/−*^ mice. Of note, as described by others and us before (Kakiyama et al., 2020; Evangelakos et al., 2021), *Cyp7b1* is tightly associated with insulin resistance and cold-exposed *Cyp7b1* ^*−/−*^ mice seem to be insulin-resistant. This might be explained by the reduced thermogenic activity and subsequent lower energy expenditure observed in *Cyp7b1* ^*−/−*^ mice, which ultimately results in the development of obesity. As LPL is activated by insulin (Sadur and Eckel, 1982) also in BAT (Mitchell et al., 1992), and LPL activation is compromised by insulin resistance (Panarotto et al., 2002), reduced LPL levels in cold-housed *Cyp7b1* ^*−/−*^ mice might result from their insulin resistance. Importantly, contrary to its regulation in adipose tissues, LPL is not regulated by insulin in the heart, where cold acclimation induces LPL levels (Cheng and Hauton, 2008). In line with comparable lipid uptake, LPL and GPIHBP1 levels were also unchanged in the hearts (Supplementary Figure S3) of cold-housed *Cyp7b1* ^−/−^ and WT mice. These data suggest that upon cold exposure, loss of *Cyp7b1* results in insulin resistance and thus impairs LPL-dependent lipoprotein processing and lipid uptake into BAT but not into the heart.

Altogether, we found that CYP7B1-derived BA are important mediators of the BAT function and impact on BAT-dependent lipoprotein processing and lipid clearance in response to cold temperatures. Mechanistically, we found that compromised lipoprotein processing and fatty acid uptake in *Cyp7b1* ^*−/−*^ mice were dependent on reduced LPL levels, which were linked to global insulin resistance (see graphical summary in Supplementary Figure S4). Our study highlights the importance of BA as modulators of BAT thermogenesis.

## Data Availability

The gene expression raw data of this article can be found online at Worthmann, Anna; Evangelakos, Ioannis (2022), “Gene Expression Liver/BAT Cyp7b1ko warm cold”, Mendeley Data, V1, doi: 10.17632/ywcpv6wwzr.1. Other raw data supporting the conclusions will be made available upon request.

## Supplementary Material

Supplementary Figure S1 Absolute concentrations of individual bile acids detected in the plasma of wild-type (WT) and *Cyp7b1* ^*−/−*^ littermates that were fed a Western-type diet (WTD) and housed for one week either at thermoneutral (30°C) or cold (6°C) environment (n=3-4/group). Data are shown as mean values ± SEM. Different letters indicate significant differences between groups (p < 0.05) determined by two-way ANOVA.

Supplementary Figure S2 Protein levels and relative quantification of pCREB and CREB **(A)** and T3 levels **(B)** in BAT of WT (n=9) and *Cyp7b1* ^*−/−*^ (n=8) littermates that were fed a WTD and housed for one week at cold (6°C) environment. Data are shown as mean values ± SEM. Different letters indicate significant differences between groups (p < 0.05) determined by two-way ANOVA.

Supplementary Figure S3 Protein levels and relative quantification of LPL and GPIHBP1 in the heart of WT (n=9) and *Cyp7b1* ^*−/−*^ (n=8) littermates that were fed a WTD and housed for one week at cold (6°C) environment. Data are shown as mean values ± SEM. Different letters indicate significant differences between groups (p < 0.05) determined by two-way ANOVA.

Supplementary Figure S4 Graphical summary.
