# Supplementary figures and images for: Cold-Induced Lipoprotein Clearance in Cyp7b1-Deficient Mice
Source: Front Cell Dev Biol. 2022 Apr 11;10:836741. doi: 10.3389/fcell.2022.836741 (PMC9038073; doi:10.3389/fcell.2022.836741)

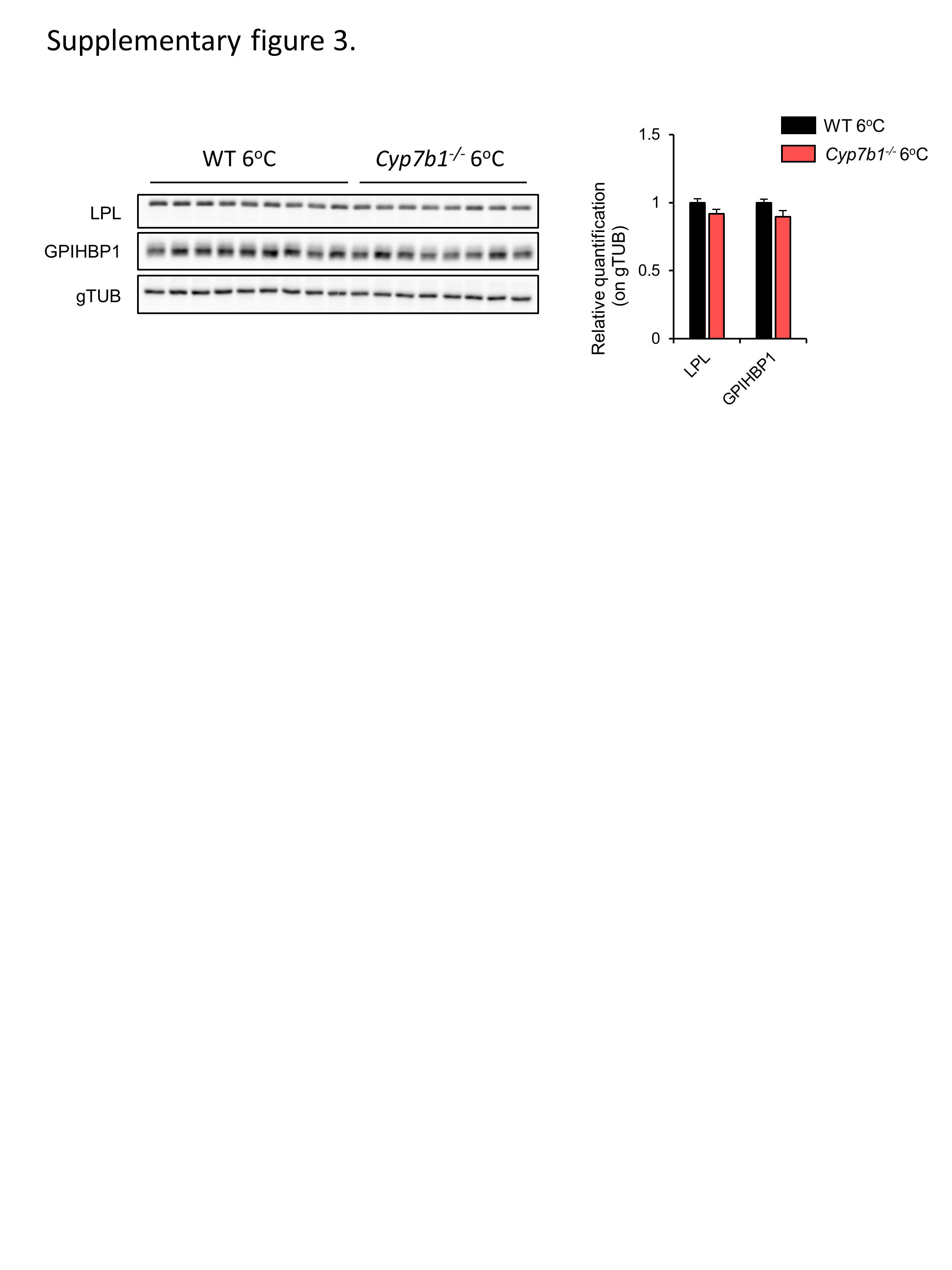

Supplement: Supplementary file 1 [file Image3.tif]

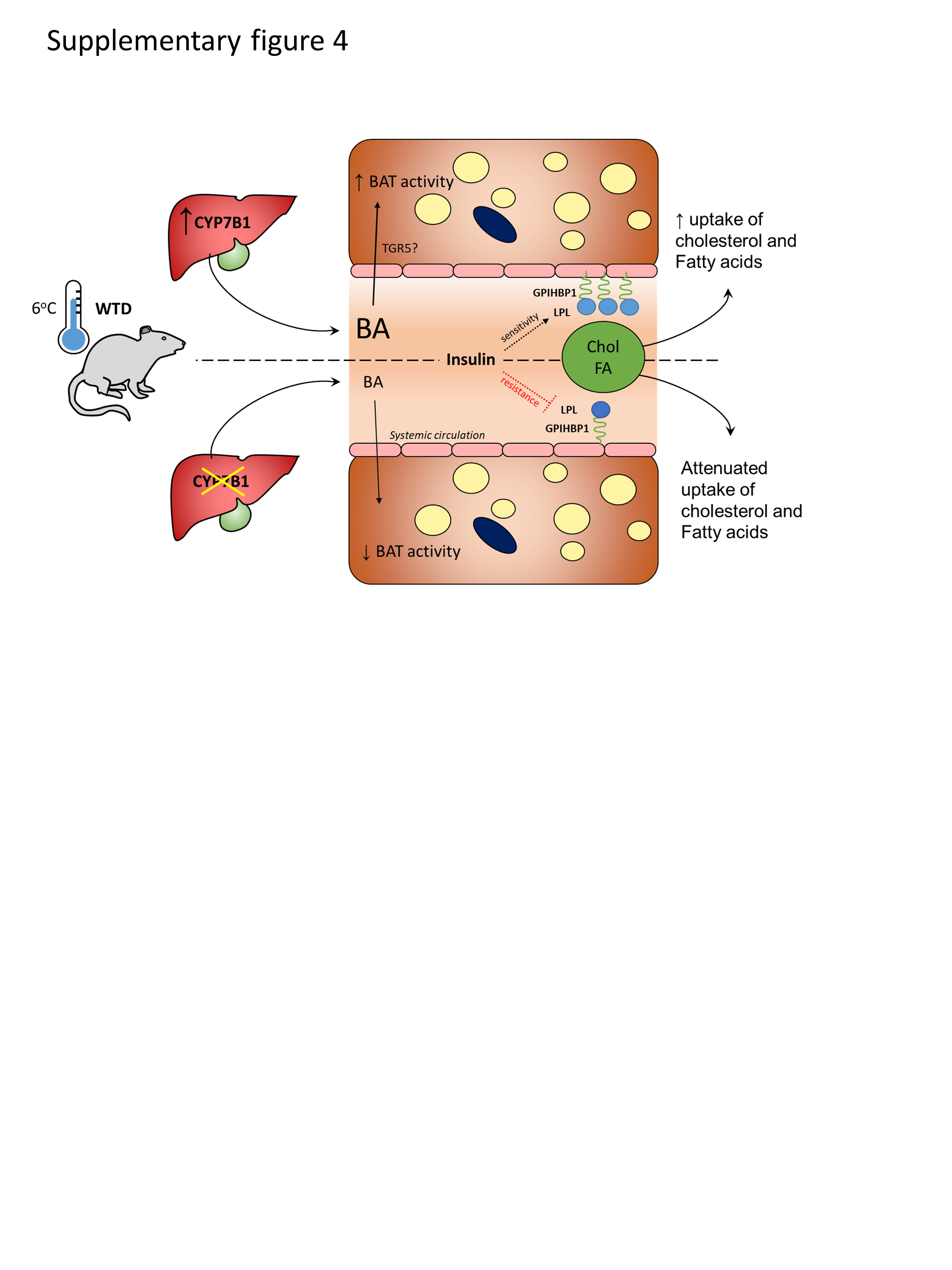

Supplement: Supplementary file 2 [file Image4.tif]

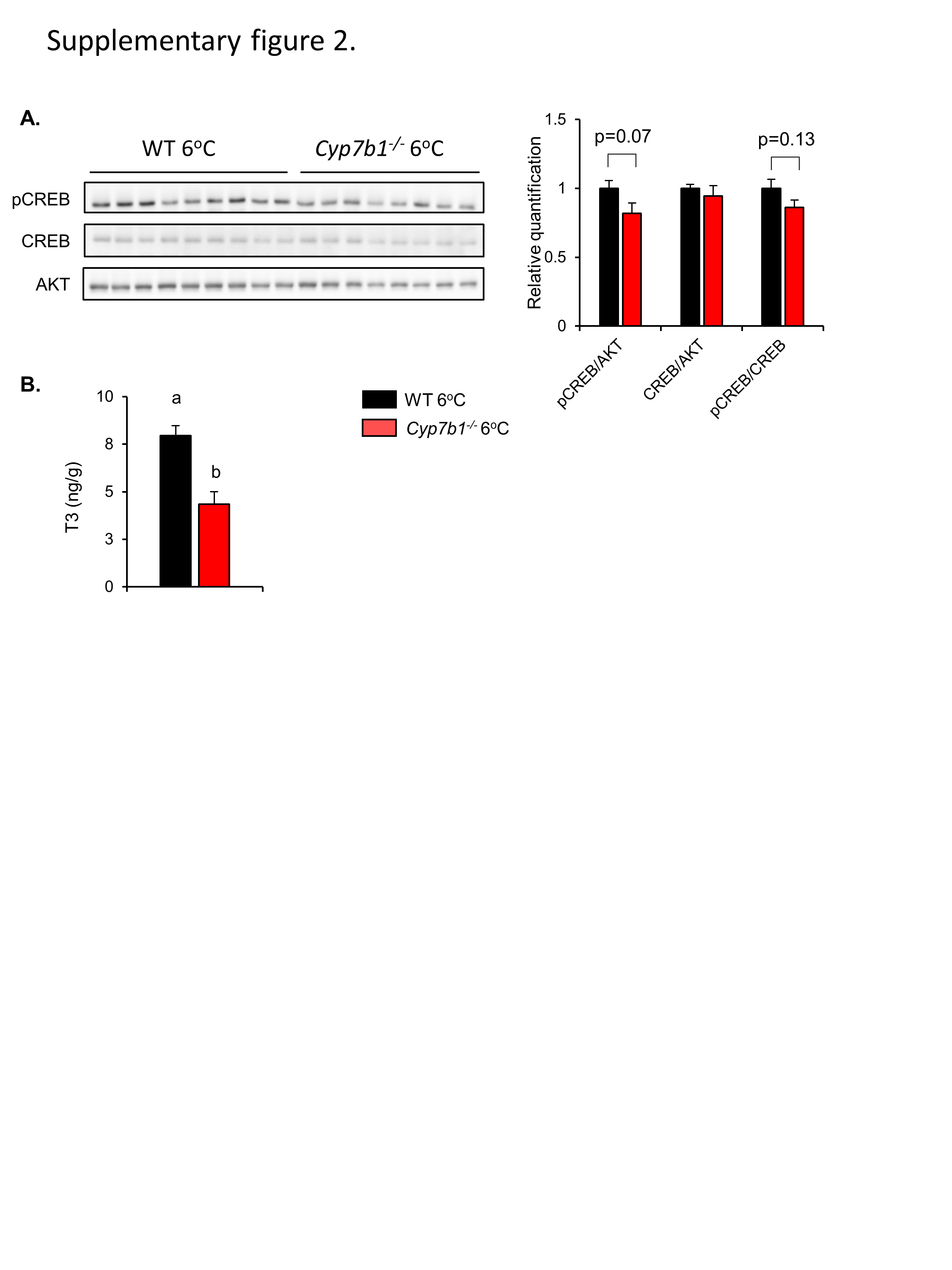

Supplement: Supplementary file 3 [file Image2.tif]

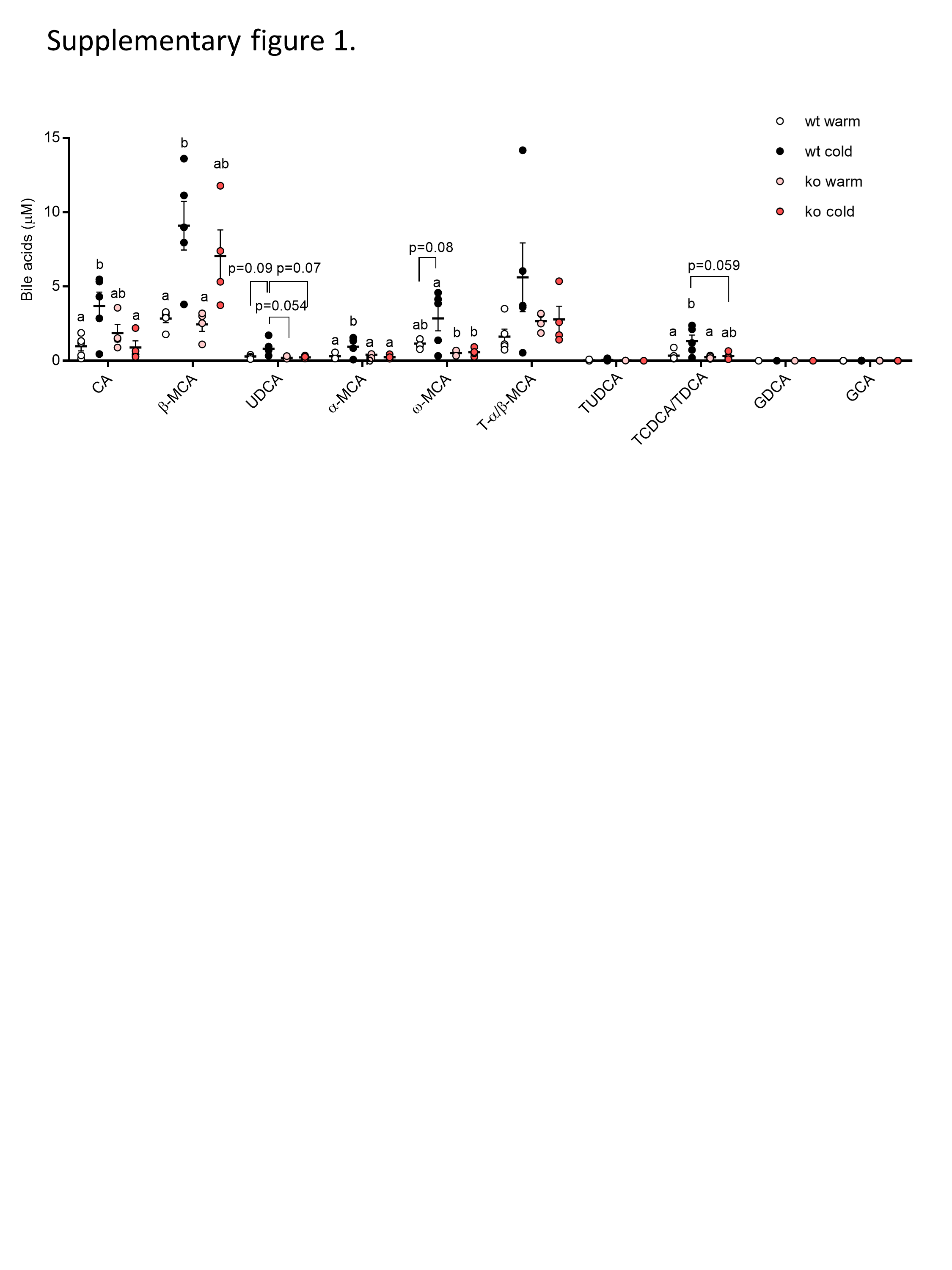

Supplement: Supplementary file 4 [file Image1.tif]
